# Correction to: “Heart failure: an update from the last years and a look at the near future” and articles listed below

**DOI:** 10.1002/ehf2.14323

**Published:** 2023-03-23

Funding section was not complete.

This should have read:

Funding: This study was supported by the Italian Ministry of Health (Ricerca Corrente) 20/1819.

The affected articles are listed in the reference list.

In addition in Riccardi et al,^1^ in paragraph “Treatment of heart failure with reduced ejection fraction”, section “Pharmacological therapies”, sub‐section “Sodium–glucose co–transporter–2 inhibitors” (page 4 out of 27) as regard the sentence “Tomasoni et al. reviewed growing evidence that support the early, upfront initiation of SGLT2is in both acute and chronic HF patients due to early benefits, with clinically meaningful reductions in clinical events that reach statistical significance within days to weeks, safety, and tolerability.” only reference 100 is correct. References 154, 342, 343 are incorrect here and they should be removed.

We apologize for this error.
